# Phosphoproteomic profiling of tumor tissues identifies HSP27 Ser82 phosphorylation as a robust marker of early ischemia

**DOI:** 10.1038/srep13660

**Published:** 2015-09-02

**Authors:** Muhammad Saddiq Zahari, Xinyan Wu, Sneha M. Pinto, Raja Sekhar Nirujogi, Min-Sik Kim, Barry Fetics, Mathew Philip, Sheri R. Barnes, Beverly Godfrey, Edward Gabrielson, Erez Nevo, Akhilesh Pandey

**Affiliations:** 1 McKusick-Nathans Institute of Genetic Medicine and Department of Biological Chemistry, Johns Hopkins University School of Medicine, Baltimore, MD 21205 USA; 2 Institute of Bioinformatics, International Tech Park, Bangalore, 560066 India; 3 Robin Medical, Inc., P.O. Box 2414, Baltimore, MD 21203, USA; 4 Charles River Discovery Research Services, 3300 Gateway Centre Boulevard, Morrisville NC 27560; 5 Department of Pathology, Johns Hopkins University School of Medicine, Baltimore, Maryland 21231, USA; 6 Department of Oncology, Johns Hopkins University School of Medicine, Baltimore, Maryland 21231, USA; 7 Adrienne Helis Malvin Medical Research Foundation, New Orleans, LA 70130, USA; 8 Diana Helis Henry Medical Research Foundation, New Orleans, LA 70130, USA

## Abstract

Delays between tissue collection and tissue fixation result in ischemia and ischemia-associated changes in protein phosphorylation levels, which can misguide the examination of signaling pathway status. To identify a biomarker that serves as a reliable indicator of ischemic changes that tumor tissues undergo, we subjected harvested xenograft tumors to room temperature for 0, 2, 10 and 30 minutes before freezing in liquid nitrogen. Multiplex TMT-labeling was conducted to achieve precise quantitation, followed by TiO_2_ phosphopeptide enrichment and high resolution mass spectrometry profiling. LC-MS/MS analyses revealed phosphorylation level changes of a number of phosphosites in the ischemic samples. The phosphorylation of one of these sites, S82 of the heat shock protein 27 kDa (HSP27), was especially abundant and consistently upregulated in tissues with delays in freezing as short as 2 minutes. In order to eliminate effects of ischemia, we employed a novel cryogenic biopsy device which begins freezing tissues *in situ* before they are excised. Using this device, we showed that the upregulation of phosphorylation of S82 on HSP27 was abrogated. We thus demonstrate that our cryogenic biopsy device can eliminate ischemia-induced phosphoproteome alterations, and measurements of S82 on HSP27 can be used as a robust marker of ischemia in tissues.

Phosphorylation of proteins is one of the main mechanisms of cellular signal transduction^1^. This process is exquisitely controlled by the action of kinases and phosphatases whose respective tasks are to add or remove phosphate groups from proteins in response to extracellular or intracellular cues. In many cases, phosphorylation at specific amino acid residues regulates the activity of the phosphorylated proteins, either activating or inhibiting their function. Reflecting the importance of protein kinases in cellular function, many kinases and phosphatases are mutated, overexpressed, hyperactivated or deleted in various cancers, thereby conferring the cancerous cells with a proliferation advantage. Accordingly, kinases commonly represent promising targets for cancer therapy through pharmacological inhibition^2^,^3^. Individualized therapy based on identification of kinase-driven signaling pathways depends on discovery of phosphorylation signals in tissues, which in turn, depends on the integrity of the tumor tissues.

As we move into the age of precision medicine to treat cancer^4^, tumor tissues are frequently evaluated for molecular alterations through genomic, transcriptomic and functional proteomic analyses, including the phosphoproteome^5^,^6^,^7^,^8^,^9^. While DNA, RNA and protein have been shown to be stable after tissue excision for an extended period of time, protein phosphorylation appears to be more labile^10^,^11^ due to the fact that tissues are biochemically active *ex vivo,* with kinase and phosphatase enzymes acting to alter proteins involved in signaling pathways^12^. In particular, a sudden loss of blood supply and deprivation of oxygen and nutrients lead cells to activate stress response pathways that decrease metabolic demands and conserve energy resources. Thus, ischemia results in global phosphoproteome changes in tissues^11^,^12^,^13^. Ischemia-induced changes in protein phosphorylation become very important when tumors tissues are studied for developing targeted therapeutic strategies^14^,^15^,^16^. As these therapies move into the clinic, the need for accurate assessment of the phosphorylation state of these kinases in patient tumors becomes critical so as to avoid misinterpretation of the tumor pathology and consequently the wrong clinical decisions for the patient.

In this study, we employed an unbiased and global mass spectrometry-based approach on mouse xenograft tumors to profile the changes of the phosphoproteome of tumors undergoing ischemia with the specific aim to identify a robust biomarker whose changes signify the ischemic condition in a tissue. We identified the heat shock protein 27 (HSP27) to be hyperphosphorylated at the serine 82 (S82) residue within a short period of ischemia, supporting other studies which have documented this site as important in ischemia. We describe the development of a novel cryogenic biopsy device that initiates freezing of tumors *in situ* prior to excision with the aim of preserving the molecular integrity of the tissues. We showed using HSP27 S82 as a surrogate biomarker that ischemia was prevented in tissues biopsied using our novel cryogenic device.

## Results and Discussion

### Tumors undergo phosphoproteomic alterations after harvest

**Figure 1:**
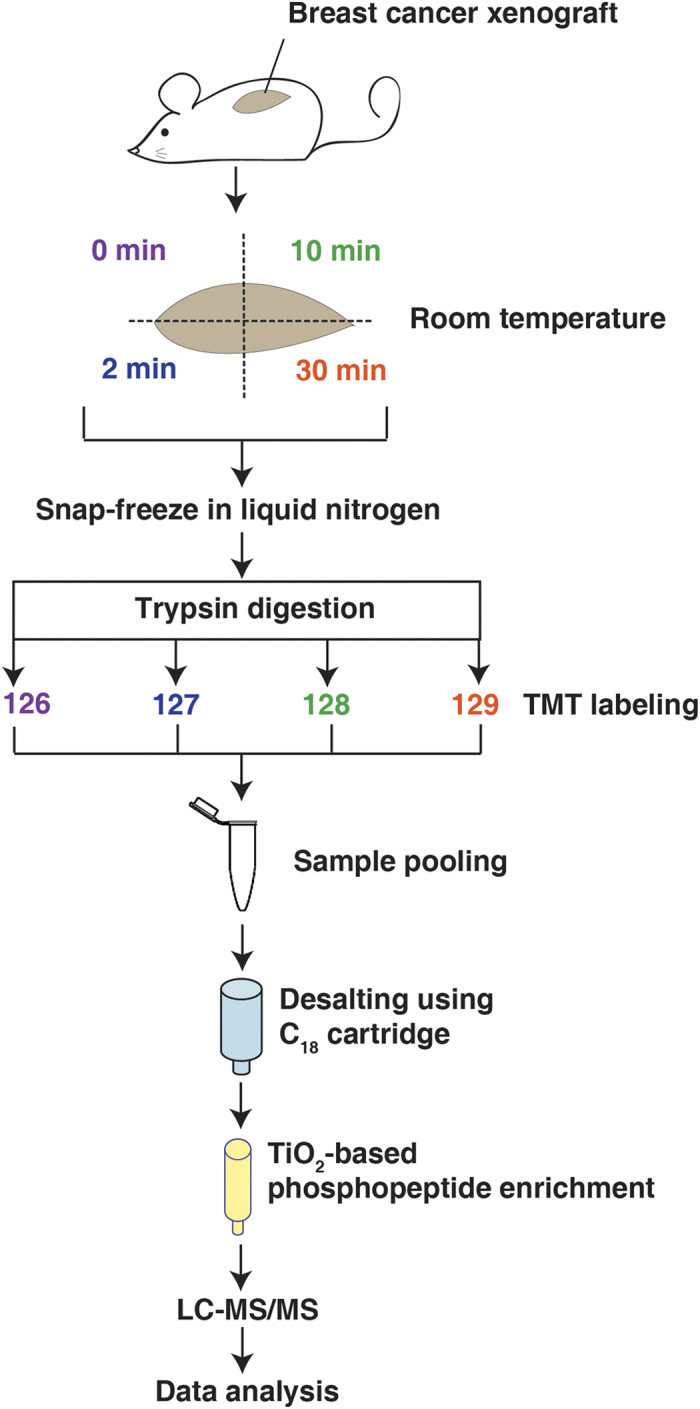
Phosphoproteomic profiling of xenograft tumors during ischemia. A schematic workflow of the strategy used to profile the phoshoproteomic changes resulting from ischemia. Whole xenograft tumors from the breast cancer cell line HCC1395 were harvested from mice and exposed to room temperature for 0, 2, 10 and 30 minutes before snap freezing in liquid nitrogen. After protein extraction and digestion with trypsin, each sample was labeled with different versions of TMT reagents followed by pooling, desalting, and enrichment using TiO_2_ beads. The enriched phosphopeptides were then analyzed on LTQ-Orbitrap Elite mass spectrometer without further fractionation. Figure drawn by M.S.Z.

In order to catalog the phosphoproteomic changes that result from ischemia, we designed an experiment in which harvested xenograft tumors were exposed to room temperature for different length of time before freezing. We used xenografts of HCC1395 breast cancer cells established in immunodeficient non-obese diabetic/gamma interferon knock-out (NSG) mice for our evaluation. When the tumors were ~1 cm in diameter, the mice were euthanized and the tumors were excised as quickly as possible. The tumors were partitioned into four equal parts, and each part was either immediately snap frozen in liquid nitrogen bath or left on the bench at room temperature for 2, 10 and 30 minutes prior to snap freezing. The tumors were then cryofractured and proteins were extracted using 4% SDS buffer and digested using trypsin. The tryptic peptides were labeled with different versions of TMT isobaric mass tags to allow for multiplexing and precise quantitation of phosphorylation levels between samples^17^. Samples were then mixed together in equal amounts and TiO_2_-based phosphopeptide enrichment was carried out. In order to identify a biomarker that is abundant and robust, we carried out a single shot LC-MS/MS analysis without further fractionation of the TMT-labeled, TiO_2_-enriched phosphopeptides (Fig. 1).

Our LC-MS/MS analysis identified 1,451 unique phosphopeptides from 774 proteins (Supplementary Tables S1 and S2). Remarkably, a number of phosphopeptides exhibited significant changes in phosphorylation levels as early as 2 minutes post-excision. For example, we observed 57 phosphopeptides whose phosphorylation levels were upregulated by at least 1.5-fold at 2 minutes compared to time 0 and this hyperphosphorylation was largely sustained thereafter. This indicates that a number of proteins have already undergone significant changes in phosphorylation levels as soon as 2 minutes post-harvest (Supplementary Table S3). Surprisingly, decreases in phosphorylation levels of proteins over the course of our experiments were less common, with only 9 phosphopeptides showing at least 33% decrease in phosphorylation levels at 2 minutes compared to time 0 (Supplementary Table S3). These findings indicate that the effects of kinase activation far outweigh protein dephosphorylation through the action of phosphatases in the first minutes of ischemia. This observation is supported by a similar study by Baker *et al.* who showed that phosphorylation of S473 of AKT in xenograft tumors levels only decreased after 20 minutes of exposure to room temperature post-excision^18^.

A large subset of phosphopeptides identified in our study however remained relatively stable, indicating that ischemia might not affect analysis of all phosphoproteins equally. Of note, no other published studies thus far have examined ischemia-induced phosphorylation changes within a window of just two minutes. We consider the phosphopeptides that underwent significant changes in phosphorylation levels to represent potential biomarkers to signify ischemia.

While this study was being concluded, another study on effects of ischemia on the proteome and phosphoproteome was published by the National Cancer Institute’s Clinical Proteomic Tumor Analysis Consortium (NCI-CPTAC). In this study, the authors used ovarian tumors from patients and patient-derived breast cancer xenografts^11^, and similar to our results, the authors found more upregulation than downregulation of peptide phosphorylation in ischemic tissues. Specifically, they found that up to 24% of the identified phosphopeptides experienced phosphorylation changes, with common ischemia-regulated proteins belonging to EGFR/MAPK signaling pathways. This study further supports our findings that the phosphoproteome undergoes changes during ischemia and warrants caution to be taken when handling resected tumor tissues and in interpreting data showing apparent activation of kinase signaling pathways.

### Phosphorylation of Serine 82 of HSP27 is induced by brief ischemia

**Figure 2:**
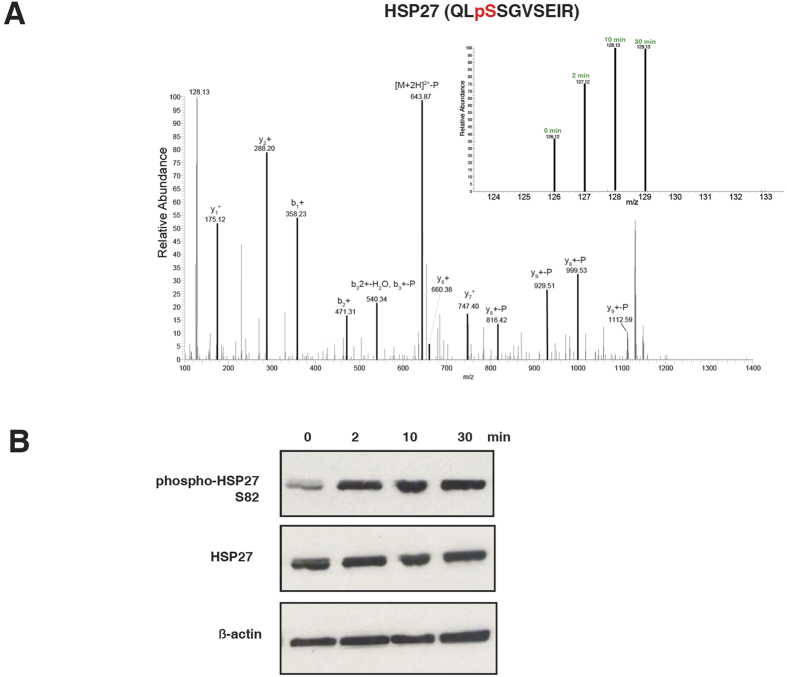
Upregulation of S82 phosphorylation of HSP27 during ischemia. (**A**) A representative MS/MS spectrum for HSP27 (QLsSGVSEIR) and the relative intensities of the TMT reporter ions for the different time points are shown on upper left. (**B**) Western blot analysis of the ischemia samples with the indicated antibodies. β-actin is used as loading control.

Examining the list of peptides whose phosphorylation underwent significant changes during ischemia (Supplementary Table S3), we found that a few of these peptides belong to proteins involved in mRNA metabolic processes, namely RBM25, SERBP1, SRRM2 and ZRANB2. Several others are kinases or kinase-interacting proteins, such as EPHA2, MAP4KP, ABI1, and TAB2. Two other proteins are involved in the unfolded protein response, namely HSP90AB1 and HSPB1. HSPB1, which is more widely known as HSP27, is of particular interest because it is a member of the chaperone proteins, whose primary functions are to provide thermotolerance, cellular protection and support of cell survival under stress conditions^19^,^20^. It has been shown that phosphorylation of HSP27 is vital for its oligomerization and interaction with specific protein targets such as cytochrome c to improve survival^21^,^22^,^23^,^24^. Correspondingly, phosphorylation of S82, which is the particular site we identified, has been shown to be important in mediating neuroprotection against ischemic neuronal injury in mouse models^25^. This site was also identified by the NCI-CPTAC group as one of the phosphorylation sites commonly upregulated in ischemic tumors^11^. Exhaustive fractionations allowed the authors to quantify over 25,000 phosphosites, and even though our study identified a much smaller number of phosphosites, HSP27 S82 phosphorylation was still observed to be upregulated (Fig. 2A). This signifies that even without reaching the highest profiling depth possible, one can infer that a tissue has experienced ischemic conditions with the hyperphosphorylation of HSP27 S82. Moreover, the corresponding phosphorylation site in pig, HSP27 S84, was found to be hyperphosphorylated in porcine muscle up to 6 hours postmortem, indicating that this site and its function in ischemia is conserved across species^26^.

To pursue HSP27 as an ischemia biomarker in greater detail, we conducted western blot analysis of the same samples using phospho-specific antibody against S82 of HSP27. We showed that the phosphorylation level is markedly increased in ischemic tissue samples as soon as 2 minutes of exposure to room temperature before snap freezing (Fig. 2B). In fact, the greatest change in phosphorylation level is seen between 0 and 2 minutes, and the hyperphosphorylation levels off thereafter and is sustained until 30 minutes. This indicates that the phosphorylation changes of this site occur rapidly during ischemia. Of note, our mass spectrometry data show that another site on HSP27, S15, also experienced sustained increase in phosphorylation levels in excised samples exposed to room temperature. Our western blot analysis using antibody against this phosphosite demonstrate elevation of phosphorylation during ischemia, providing support of the robustness of HSP27 as an ischemia marker (Supplementary Fig. S1).

### A novel cryogenic biopsy device can reduce ischemia-induced alterations

**Figure 3:**
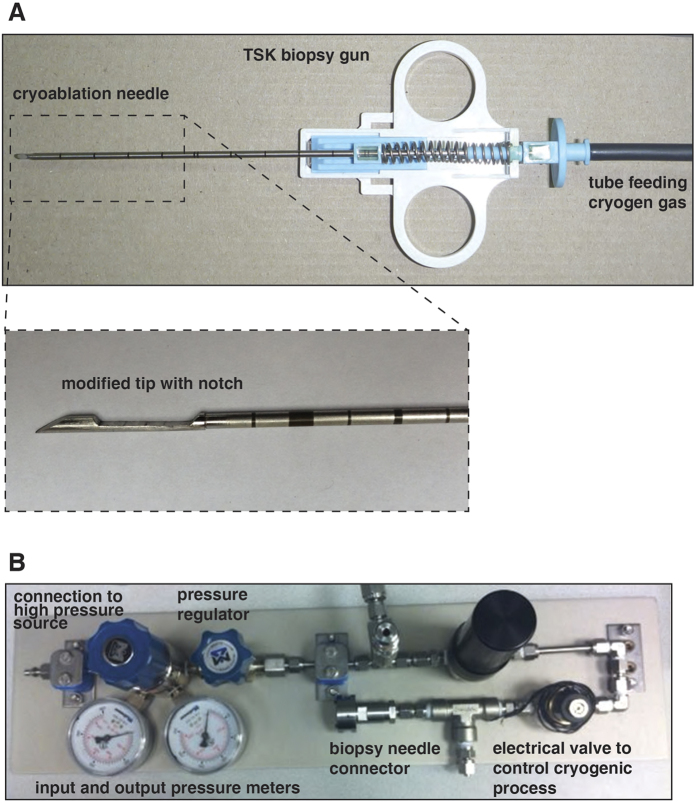
Development of novel cryogenic biopsy device. (**A**) A picture of the cryogenic biopsy device made using a conventional TSK biopsy gun with a tube feeding cryogen gas and a modified cryoablation needle (inset). (**B**) A picture of the simple cryogenic system containing manual pressure regulator to set the input pressure to the needle, a safety valve, and a solenoid for computer controlled operation

Storage and tissue handling of surgical tumor or core needle biopsy specimen have been recognized as critical steps that can potentially affect reproducibility and comparability of molecular endpoints^27^. We have shown in this study that tumor tissue ischemia time could rapidly affect protein phosphorylation after surgical extirpation. In a busy clinical setting, variability exists between the time of tissue excision to time of tissue freezing/fixation for pathological examination and analyses. Consequently, these variations could significantly affect the accuracy of analytical data and misrepresent the situations of cells *in vivo*. To maximally eliminate the ischemia effects, we have developed a novel cryogenic core needle biopsy device that begins freezing tumors *in situ*. The device is composed of a modified cryoablation needle (IceSeed, Galil Medical, Inc., Arden Hills, MN) and a cutting cannula of a standard biopsy needle (Stericut 14G, TSK Laboratory, Japan) (Fig. 3A). The tip of the cryoablation needle has been replaced with a longer tip having a side notch for the biopsy sample (Fig. 3A). The modified needle replaces the stylet of the Stericut biopsy needle. The cryogen substance is carbon dioxide at pressure range of 400–800 psi. A simple cryogenic system has been built containing manual pressure regulator to set the input pressure to the needle, a safety valve, and a solenoid for computer controlled operation (Fig. 3B). The insertion of the stylet into the tumor fills the notch with a small sample of the tumor tissue, and the cryogenic module positioned underneath the sample notch rapidly freezes the sample. After the cannula is pushed forward to dissociate the sample from the surrounding tissue, the stylet is extracted from the tumor while the cryogenic module maintains the sample in a frozen state to prevent any changes in the biomolecular profile of the cells. Using this cryogenic biopsy device in the clinical setting could eliminate variability of time from tissue excision to tissue fixation/freezing, thus avoiding phosphoproteome changes resulting from ischemia and increasing confidence in results of molecular analyses done on these tissues.

### Use of novel cryogenic biopsy device prevents hyperphosphorylation of S82 of HSP27

**Figure 4:**
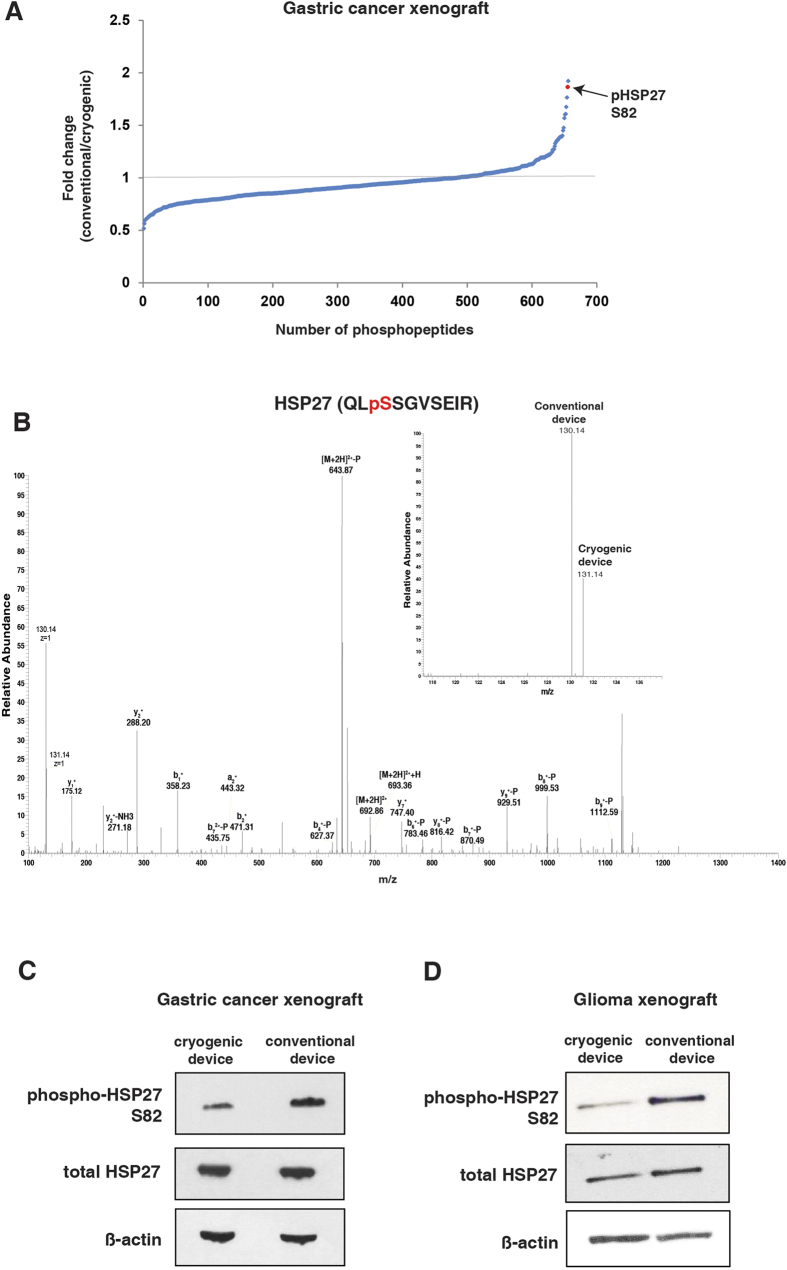
Prevention of hyperphosphorylation of HSP27 S82 by the novel cryogenic biopsy device. (**A**) A distribution of ratios of all phosphopeptides identified in the mass spectrometry analysis of NCI-N87 gastric xenograft tumors biopsied using conventional/cryogenic device. (**B**) A representative MS/MS spectrum for HSP27 (QLsSGVSEIR) and the relative intensities of the TMT reporter ions for the different biopsy devices are shown on upper left (**C**,**D**) Western blot analysis of the NCI-N87 gastric xenograft tumor and U87MG glioma xenograft tumor biopsied with the cryogenic and conventional devices using the indicated antibodies. β-actin is used as loading control.

In order to evaluate the efficiency of the cryogenic device in prevention of tissue ischemia, we employed an unbiased mass spectrometry-based approach to globally examine the protein phosphorylation changes in xenograft tumors in mice. Here, we generated the xenografts using the gastric cancer cell line NCI-N87. We utilized another tumor type in order to allow us to identify a biomarker that is robust and consistent across tumor types. We performed biopsy of the tumors with either the cryogenic device or the conventional biopsy device. Both samples were snap-frozen 2 minutes after the biopsy was extracted from the tumor. During this period, the cryogenic device maintained the tissue in frozen state whereas the conventional device biopsy was left exposed to room temperature. The samples were then processed for mass spectrometry analysis as per previous round of experiment (Supplementary Fig. S2). Here, we identified 656 phosphopeptides from 404 proteins (Supplementary Tables S4 and S5), of which 76 were found to be significantly hyperphosphorylated in the sample biopsied using the conventional device compared to the cryogenic sample. Examining the list of hyperphosphorylated peptides, we identified a number of phosphopeptides that were also upregulated in the profiling experiment we carried out earlier, indicating that the gastric tumors biopsied with the conventional device experienced similar ischemic conditions (Table 1). Among these, HSP27 S82 was found to be one of the most hyperphosphorylated peptides in the conventional sample (Fig. 4A,B). Thus this shows that HSP27 S82 is a particularly reliable biomarker to detect the ischemic conditions that a tissue is experiencing.

We then confirmed our mass spectrometry observation with a western blot analysis on the same samples using a phospho-specific antibody against S82 of HSP27 (Fig. 4C). To further evaluate the robustness of this site as an ischemia biomarker, we generated another xenograft tumor using the glioma cell line U87MG in a similar fashion as the gastric cancer xenografts. We performed biopsy of these tumors using the cryogenic device and conventional device as the previous experiment. We then carried out western blot analysis to compare the phosphorylation of S82 of HSP27 between these two samples. Consistent with the previous experiment, we found that S82 of HSP27 is hyperphosphorylated in the conventional sample compared to the cryogenic sample (Fig. 4D). This shows that S82 hyperphosphorylation of HSP27 is a robust biomarker for ischemia and this hyperphosphorylation, and by extension ischemia, could be avoided using our novel cryogenic biopsy device.

In summary, our study demonstrates that tumor tissues undergo rapid phosphoproteome changes after excision, even in a time window as short as 2 minutes. We have found across three tumor types that S82 of HSP27 is hyperphosphorylated during ischemia, and hence providing us with a rapid and robust ischemia biomarker. We further developed a novel cryogenic biopsy device and showed that using this device, hyperphosphorylation of HSP27 S82 could be prevented. Single reaction monitoring (SRM) assays could also be developed to monitor the levels of other ischemia markers identified from our study of which reliable phospho-specific antibodies are not available.

## Methods

### Generation and preparation of breast cancer-derived xenograft tumors in immunodeficient mice

The triple negative breast cancer cell line HCC1395 (ATCC) was grown *in vitro* prior to the xenograft study. 1 × 10^6^ cells were subcutaneously injected per site into immunocompromised nod-SCID gamma (NSG) mice. Tumors were allowed to develop for about 3–4 weeks until they were ~1 cm in diameter. Animals were housed in the Johns Hopkins University animal facility and study was conducted according to approved protocols by the IRB. The mice were euthanized using an overdose of isoflurane (Baxter). The tumors were excised as quickly as possible and were left exposed on the bench at room temperature for 0, 2, 10 and 30 minutes, respectively, followed by snap freezing in liquid nitrogen bath. Experiments were performed on two tumors, with one of the tumor sets divided into two parts which were then separately processed and analyzed as replicates.

### Generation and biopsy of gastric and glioma-derived xenograft tumors in immunodeficient mice

U87MG human glioma-derived tumors were maintained through subcutaneous transplantation on mice. Briefly, U87MG xenografts of approximately 1000 mm^3^ in size were excised, cut to 1 mm^3^ fragments, and implanted into female athymic nude mice (Crl:NU(Ncr)-*Foxn1*^*nu*^, Charles River) using a trocar. Tumors grew to the target range of 1000 mm^3^–1500 mm^3^ 22 days after implant and this procedure was then repeated. 1 × 10^7^ NCI-N87 tumor cells containing 50% Matrigel™ (BD Biosciences) were implanted into the right flank of female Fox Chase SCID^®^ Mice (CB17/Icr-*Prkdc*^*scid*^/IcrIcoCrl, Charles River). Tumors grew to a target range of 600 mm^3^–800 mm^3^ fifty-seven days after implant. Animals were housed in animal facility of Charles River Discovery Research Services, North Carolina, which specifically complies with the recommendations of the *Guide for Care and Use of Laboratory Animals* with respect to restraint, husbandry, surgical procedures, feed and fluid regulation, and veterinary care. Animals were placed under isoflurane anesthesia prior to sampling and were euthanized immediately after sampling. All tumors were sampled once by conventional biopsy needle (Semi-automatic biopsy gun, *In Vivo* Corp., Gainesville, FL) and once by the experimental cryobiopsy device. The cryoablation needle is based on a clinical cryoablation needle (IceSeed) by Galil Medical (Arden Hills, MN) with modified tip as shown in Fig. 3. The needle is connected to the cryogenic system by a coaxial, double-lumen tube that carries the inflow gas (CO_2_) in the central tube and the outflow gas (after expansion) in the outer tube. The high-pressure inflow gas is fed into the needle where it expands in a Joule-Thomson process that extracts heat from the surrounding and causes a steep decrease in the temperature of the needle tip. The outflow gas exits the tube to the surrounding air at the connecting point to the cryogenic system. The cryoablation needle serves as the stylet of the biopsy device, with the cannula surrounding it. After the needle tip is inserted into the tumor, the cooling process freezes the tissue around the tip, and the cannula is pushed forward by the spring mechanism of the biopsy device and cuts the frozen sample inside the notch, as in standard core biopsy devices. The biopsy needles were inserted into the tumor under direct visual guidance until the biopsy notch was within the tumor. The flow of the cryogenic agent was started and the biopsy cannula was shot after 10 seconds, which allowed the tissue within the biopsy notch to be frozen before being cut. All samples were incubated in room temperature for 2 minutes, with the cryobiopsy samples being maintained frozen within the cryobiopsy device. Following the incubation time the samples were snap frozen in liquid nitrogen. Samples biopsied from four tumors were pooled for subsequent sample processing and mass spectrometry analysis.

### Protein extraction, digestion and labeling

The frozen tumors were ground using mortar and pestle that were precooled in liquid nitrogen and placed on dry ice. The pulverized powder was then transferred into 4% SDS buffer. The lysates were then sonicated to ensure complete lysis. The lysates were precleared by centrifugation at 16,000 g at 4 °C. Protein estimation was carried out using BCA assay (Pierce). 500 μg proteins from each time point was reduced with 5 mM dithiothreitol (Sigma) at 60 °C for 30 minutes and alkylated with 10 mM iodoacetamide (Sigma) for 10 minutes in the dark. The volume for each sample was then enlarged with 10 mM triethylammonium bicarbonate (TEABC) (Sigma) to 40 times the original volume to reduce the SDS composition. The proteins were then concentrated using 10 kDa Amicon Ultra centrifugal filters (Millipore). 200 μg of protein from each sample was then digested overnight at 37 °C using sequencing grade trypsin (1:40) (Promega). Tryptic peptides were labeled with 4-plex isobaric tandem mass tags (TMT) (Thermo Scientific) according to manufacturer’s instructions. Different TMT Label Reagents were added to each of the sample from each time point. Labeling reaction was carried out for 1 hour at room temperature. The reaction was quenched with 5% hydroxylamine (Thermo Scientific). The digested and labeled peptides from the different time points were pooled and desalted with 5 mg C18 SEP-PAK light reversed phase column (Waters).

### TiO_2_ enrichment

Phosphopeptides were enriched with TiO_2_-based phosphopeptide enrichment as described earlier^28^. Briefly, TiO_2_ beads (GL Sciences, Japan) were incubated with DHB solution (80% acetonitrile (ACN) (J.T.Baker), 1% trifluoroacetic acid (TFA) (Fisher), 3% 2,5-dihydroxybenzoic acid (DHB) (Sigma) for 15 minutes at room temperature. Each sample was resuspended in DHB solution and incubated with pretreated TiO_2_ beads (TiO_2_:peptide ratio of 1:1) for 1 hour. Phosphopeptide-bound TiO2 beads were washed three times with DHB solution and twice with 40% ACN. Peptides were eluted three times with 40 μl of 2% ammonia into 10 μl of 4% TFA.

### LC-MS/MS Analysis

Enriched phosphopeptides were analyzed on LTQ-Orbitrap Elite mass spectrometer (Thermo Scientific, San Jose, CA, USA) interfaced with Easy-nanoLC II nanoflow liquid chromatography system (Thermo Scientific, Odense, Southern Denmark). The peptides from each fraction were reconstituted in 0.1% formic acid and loaded on a pre-column (75 μm × 2 cm) packed in-house with magic C18 AQ (MichromBioresources, Auburn, CA, USA) 5 μ particle and 100 Å pore size at flow rate of 5 μl per minute. Peptides were resolved at 250 nl/min flow rate using a linear gradient of 10 to 35% solvent B (0.1% formic acid in 95% acetonitrile) over 95 minutes on an analytical column, 75 μm × 25 cm, 5 μ particle and 100 Å pore size packed using nitrogen pressure cell at 2,500 psi and was fitted on flex ion source that was operated at 2.0 kV voltage. Mass spectrometry analysis was carried out in a data dependent manner with a full scans in the range of m/z 350 to 2000. Both MS and MS/MS were acquired and measured using Orbitrap mass analyzer. Full MS scans were measured at a resolution of 120, 000 at m/z 400. Fifteen most intense precursor ions were selected for MS/MS and were fragmented using higher-energy collisional dissociation (HCD) method and detected at a mass resolution of 30,000 at m/z 400. Automatic gain control for full MS was set to 1 million ions and for MS/MS was set to 0.05 million ions with a maximum ion injection time of 100 and 200 ms respectively. Dynamic exclusion was set to 30 sec and singly charged ions were rejected. Internal calibration was carried out using lock mass option (m/z 445.1200025) from ambient air.

### Data analysis

Proteome Discoverer (v 1.4; Thermo Fisher Scientific) suite was used for quantitation and identification. The tandem mass spectrometry data were searched using Mascot (Version 2.2.0) and SEQUEST search algorithms against a Human RefSeq database (v 59 containing 33,249 entries) supplemented with frequently observed contaminants. The search parameters used were as follows: a) trypsin as a proteolytic enzyme (with up to two missed cleavages) b) peptide mass error tolerance of 10 ppm; c) fragment mass error tolerance of 0.05 Da; d) Carbamidomethylation of cysteine (+57.02146 Da) and TMT tags (+229.162932 Da) on lysine residue and peptide N-termini as fixed modification and oxidation of methionine (+15.99492 Da) and phosphorylation (+79.96633 Da) of Serine, Threonine and Tyrosine residues as variable modifications. The probability of phosphorylation for each Ser/Thr/Tyr site on each peptide was calculated by the PhosphoRS algorithm^29^. The relative ratio for each phosphopeptide was calculated by dividing the intensity of each phosphorylated peptide over the intensity of the corresponding peptide. Sequest peptide data were extracted with 1% false discovery rate (FDR) threshold.

### Western blot analysis

Equal amounts of lysate (60 μg) were separated by sodium dodecyl sulphate-polyacrylamide gel electrophoresis in 4–12% NuPAGE Bis-Tris Precast Gels (Novex, Life Technologies Corporation) and transferred onto nitrocellulose membranes (Whatman, GE Healthcare, Life Science, Pittsburgh, PA, USA) using a semi-dry transfer unit Hoefer TE 70 (Amersham Bioscience). The membranes were blocked with 5% low-fat milk dissolved in phosphate buffered saline containing 0.05% Tween (Sigma) (PBST) for 1 h at room temperature and incubated overnight at 4 °C with primary antibodies. Antibodies used were phospho-HSP27 S82 and S15 (Cell Signaling Technology), HSP27 (Santa Cruz) and β-actin (Sigma-Aldrich). After washing with PBST three times each for 10 min, the membranes were further incubated with the corresponding horseradish peroxidase-conjugated secondary antibodies (GE Healthcare) for 1 h at room temperature. After washing with PBST three times each for 5 min, antibody-bound protein bands were detected with ECL Western Blotting Detection Reagents (RPN 2106V1 and RPN2106V2, GE Healthcare Life Sciences, Pittsburgh, PA, USA) and developed on Amersham Hyperfilm ECL autoradiography film (GE Healthcare Life Sciences, Pittsburgh, PA, USA).

## Additional Information

**How to cite this article**: Zahari, M. S. *et al.* Phosphoproteomic profiling of tumor tissues identifies HSP27 Ser82 phosphorylation as a robust marker of early ischemia. *Sci. Rep.* **5**, 13660; doi: 10.1038/srep13660 (2015).

## Supplementary Material

Supplementary Figures

Supplementary Table S1

Supplementary Table S2

Supplementary Table S3

Supplementary Table S4

Supplementary Table S5

## Figures and Tables

**Table 1:** A list of phosphosites with upregulation of phosphorylation in both the profiling experiment at 2 vs 0 minutes and the biopsy experiment comparing the conventional to the cryogenic device.

Gene Symbol	Protein	Phosphosite	2/0 min (fold change)	conventional/cryogenic (fold change)
*HSPB1*	heat shock 27 kDa protein 1	S82	1.7	1.9
*EIF4H*	eukaryotic translation initiation factor 4H	S24	1.3	1.8
*PRKRA*	protein kinase, interferon-inducible double stranded RNA dependent activator	S18	1.4	1.5
*LMO7*	LIM domain 7	S1695, S1697	1.3	1.3
*PDLIM5*	PDZ and LIM domain 5	S228	1.5	1.6
*PNN*	pinin, desmosome associated protein	S66	1.3	1.4
