# Supplementary material for: Phosphoproteomic profiling of tumor tissues identifies HSP27 Ser82 phosphorylation as a robust marker of early ischemia
Source: Sci Rep. 2015 Sep 2;5:13660. doi: 10.1038/srep13660 (PMC4557083; doi:10.1038/srep13660)
Supplement: Supplementary Figures [file srep13660-s1.pdf]

Supplementary Information

Phosphoproteomic profiling of tumor tissues identifies HSP27 Ser82 phosphorylation as a robust marker of early ischemia

Muhammad Saddiq Zahari[1], Xinyan Wu[1], Sneha M. Pinto2, Raja Sekhar Nirujogi[2], Min-Sik Kim[1], Barry J. Fetics[3], Mathew Philip[3], Sheri R. Barnes[4], Beverly Godfrey[4], Edward Gabrielson[5,6], Erez Nevo[3], Akhilesh Pandey[1,5,6,7,8]

[1]McKusick-Nathans Institute of Genetic Medicine and Department of Biological Chemistry, Johns Hopkins University School of Medicine, Baltimore, MD 21205 USA, [2]Institute of Bioinformatics, International Tech Park, Bangalore, 560066 India, [3]Robin Medical, Inc., P.O.Box 2414, Baltimore, MD 21203, USA, [4]Charles River Discovery Research Services, 3300 Gateway Centre Boulevard, Morrisville NC 27560, [5]Department of Pathology, [6]Department of Oncology, Johns Hopkins University School of Medicine, Baltimore, Maryland 21231, USA
[7]Adrienne Helis Malvin Medical Research Foundation, [8]Diana Helis Henry Medical Research Foundation, New Orleans, LA 70130, USA

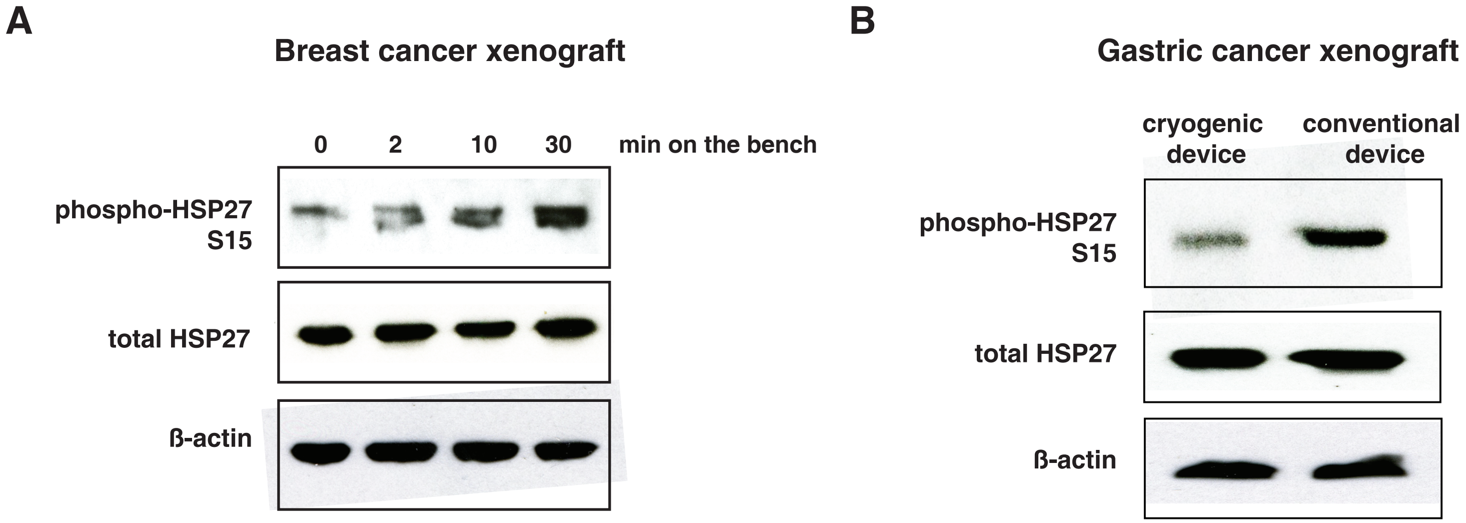

Supplementary Fig. S1: Upregulation of HSP27 S15 phosphorylation in ischemia. Western blot analysis using the indicated antibodies on **(A)** HCC1395 breast cancer-derived xenograft tumors subjected to room temperature at 0, 2, 10, and 30 minutes before snap freezing in liquid nitrogen, **(B)** NCI-N87 gastric cancer-derived xenograft tumors biopsied using the cryogenic biopsy device or the conventional biopsy device. β-actin serves as loading control.

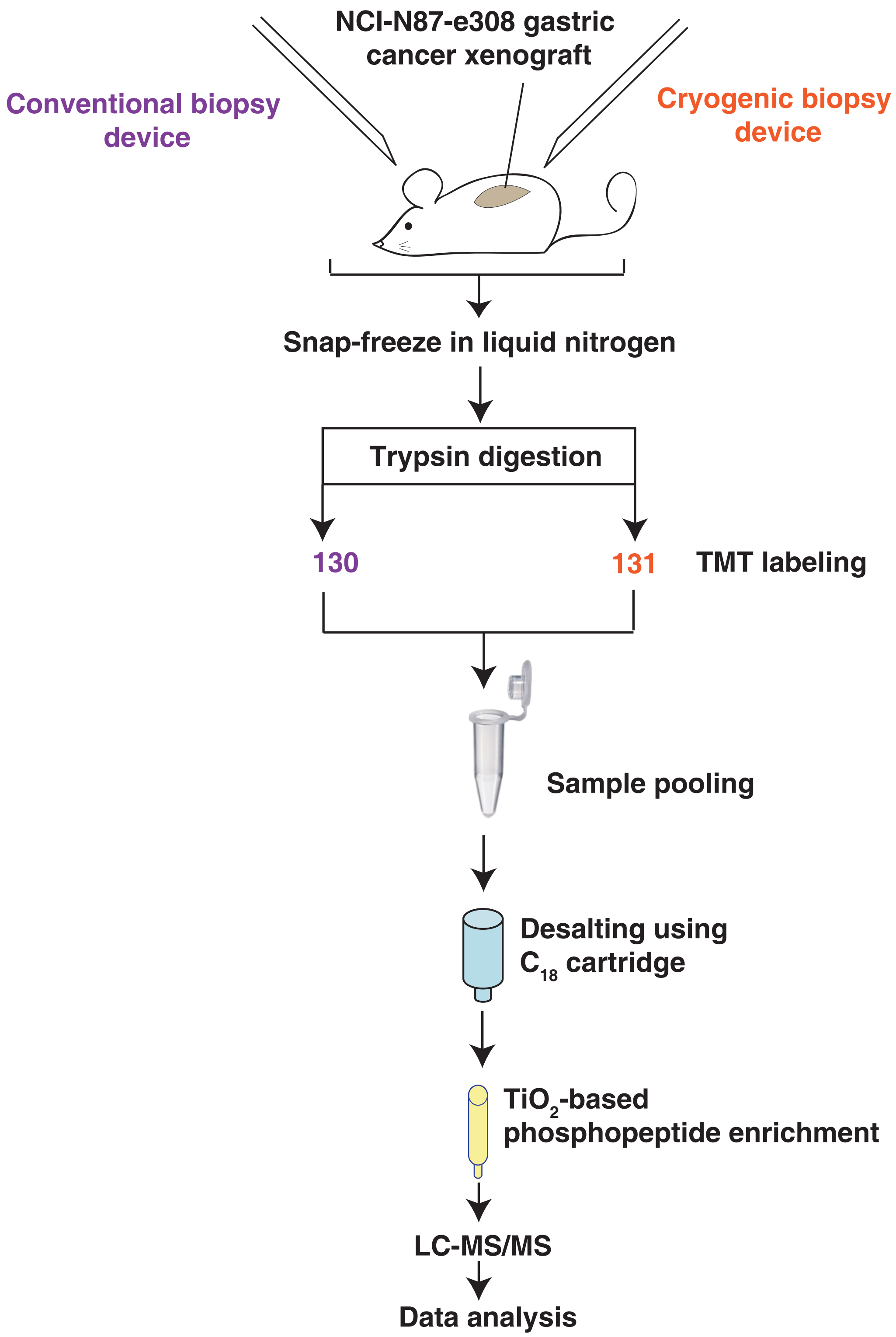

Supplementary Fig. S2. Phosphoproteomic profiling of tumor samples biopsied using the novel cryogenic biopsy device. A schematic workflow of the strategy used to profile the phoshoproteomic changes found in tumor samples biopsied using the conventional biopsy device vs the cryogenic biopsy device.
